# Pre-hospital management, procedural performance and outcomes for primary percutaneous coronary intervention in ST-elevation myocardial infarction in the Netherlands: Insights from the Dutch cohort of the APPOSITION-III trial

**DOI:** 10.1007/s12471-016-0891-x

**Published:** 2016-08-31

**Authors:** N. S. Vos, G. Amoroso, M. J. Grundeken, A. J. J. Ijsselmuiden, R. J. M. van Geuns, R. Spaargaren, J. G. P. Tijssen, K. T. Koch

**Affiliations:** 1 Onze Lieve Vrouwe Gasthuis, Amsterdam, The Netherlands; 2 Academic Medical Center, Amsterdam, The Netherlands; 3 Albert Schweitzer Hospital, Dordrecht, The Netherlands; 4 Erasmus Medical Center, Rotterdam, The Netherlands; 5 STENTYS S.A., Paris, France

**Keywords:** ST-elevation myocardial infarction, Primary percutaneous coronary intervention

## Abstract

**Aim:**

The aim of this study was to achieve useful insights into pre-hospital management and procedural performance for ST-elevation myocardial infarction (STEMI) in the Netherlands by extrapolating patient characteristics, and procedural and clinical outcomes of the Dutch patient cohort from the APPOSITION-III trial.

**Methods:**

This is a retrospective analysis from the APPOSITION-III trial with respect to the geographical borders of STEMI management. The APPOSITION-III trial was a European registry for the use of the STENTYS self-expandable stent in STEMI patients undergoing primary percutaneous coronary intervention (PPCI). 965 Patients were enrolled mainly in the Netherlands (*n* = 420, 43.5 % of the overall study population), Germany (*n* = 165) and France (*n* = 131). The data from the Dutch cohort were compared with both the overall study population, and the French and German cohorts, respectively, as well as the European Society of Cardiology (ESC) STEMI guidelines.

**Results:**

In this trial there was a wide inter-country variation on symptom-to-balloon time, 165 minutes (120–318) in the Netherlands, 270 minutes (180–650) in Germany and 360 minutes (120–480) in France, respectively. In general, a preload of dual antiplatelet therapy (DAPT) combined with heparin was more often performed in the Dutch and French cohort than in the German cohort. DAPT at discharge was high across the whole APPOSITION-III population. No important differences were seen between the different groups according to the endpoints major adverse cardiac event and stent thrombosis.

**Conclusion:**

In the Dutch cohort of an European multicentre STEMI study (APPOSITION-III trial), the performance in terms of symptom-to-balloon time, and pre-, peri- and post-procedural medical treatment is in line with the recommendations of ESC STEMI guidelines.

## Introduction

Primary percutaneous coronary intervention (PPCI) is the recommended treatment for ST-elevation myocardial infarction (STEMI) according to the most recent European Society of Cardiology (ESC) guidelines [1]. The ESC guidelines also make recommendations on appropriateness (symptom-to-balloon time) and standards (concomitant medications) for performing PPCI in STEMI patients [1].

To comply with the ESC guidelines, most European countries have developed national and/or regional networks and protocols for PPCI, and perform quality controls throughout registries. A good example is the Swedish Register of Information and Knowledge about Swedish Heart Intensive Care Admission (RIKS-HIA) [2].

Although the Netherlands was one of the first countries to perform PPCI [3], experiences with local quality assessments are sparse and there is no Dutch national registry to assess clinical performance in STEMI treatment [4, 5].

In order to achieve useful insights into pre-hospital management and procedural performance for STEMI in the Netherlands, we extrapolated patient characteristics and clinical outcomes of the Dutch patient cohort from a large post-market STEMI study, the APPOSITION-III trial [6], using the overall population and ESC STEMI guidelines as reference.

## Methods

This is a retrospective analysis from the APPOSITION-III trial with respect to the geographical borders of STEMI management. The APPOSITION-III trial was a real-life, prospective, multicentre, European, one-arm, post-market registry for the use of the STENTYS self-expandable stent (STENTYS S.A., Paris, France) in STEMI patients undergoing PPCI. The design and results of the APPOSITION-III trial have been published in detail previously [6]. This original manuscript provides information about the inclusion and exclusion criteria and study population flow chart. Briefly, patients were eligible for inclusion in the study if they presented with a STEMI caused by a de novo stenosis in a native coronary artery. The primary endpoint was the composite of major adverse cardiac events (MACE) at one-year post-procedure. A total of 965 patients were enrolled in 51 hospitals in 14 European countries, mainly in the Netherlands (*n* = 420, 43.5 % of the overall study population, see appendix A for participating centres in the Netherlands). The remaining patients were enrolled in Germany (*n* = 165), France (*n* = 131), and 11 other European countries. The data from the Dutch cohort were compared with both the overall study population, and the French and German cohorts, respectively.

### Statistical analysis

Continuous variables are presented as mean (± standard deviation) if normally distributed or median (interquartile range) in case of not-normal distribution. Categorical variables are presented as frequencies and percentages. Comparisons between groups were performed using one-way ANOVA (normal distributed continuous variables), Kruskal-Wallis (not-normal distributed continuous variables) or Chi-square test (categorical variables), where appropriate. The cumulative event rates in the Dutch subgroup vs. the French and German cohorts were compared using the log-rank test. A *p*-value of ≤0.05 is considered statistically significant. The statistical analyses were performed at the Academic Medical Center (Amsterdam, the Netherlands) using the SPSS software package (version 19.0, IBM, Chicago, IL, USA).

## Results

### Baseline demographics and patient characteristics

The baseline characteristics of the studied groups are summarised in Table 1. There were no differences in mean age and gender. The cardiovascular risk profile of the Dutch patients was similar to the overall population, although the incidence of some of the risk factors was lower compared with the German cohort.

**Table 1:** Baseline demographics and patient characteristics

Variables	Netherlands	Germany	France	*p*-value	Total study population
	*n = 420*	*n* = 165	*n* = 131		*n* = 965
Age (years)	59 ± 12	62 ± 12	59 ± 13	0.083	60 ± 12
Male gender (%)	317 (76)	125 (76)	107 (82)	0.325	743 (77)
Diabetes mellitus (%)	52 (12)	37 (22)	18 (14)	0.008	140 (15)
Hypertension (%)	161 (38)	116 (70)	51 (39)	<0.0001	456 (47)
Hypercholesterolaemia (%)	120 (29)	93 (56)	56 (43)	<0.0001	404 (42)
Current smoking (%)	240 (57)	83 (50)	79 (60)	0.185	539 (56)
Family history of CAD (%)	199 (47)	46 (28)	38 (29)	<0.0001	360 (37)
Previous MI (%)	23 (5.5)	10 (6.1)	2 (1.5)	0.003	46 (4.8)
Previous PCI (%)	28 (6.7)	11 (6.7)	3 (2.3)	0.156	54 (5.6)
Previous CABG (%)	2 (0.5)	0	1 (0.8)	0.577	3 (0.3)
Previous stroke (%)	14 (3.3)	2 (1.2)	6 (4.6)	0.085	25 (2.6)

*CAD* coronary artery disease, *MI* myocardial infarction, *PCI* percutaneous coronary intervention, *CABG* coronary artery bypass graft

### Peri-procedural characteristics

**Fig. 1:**
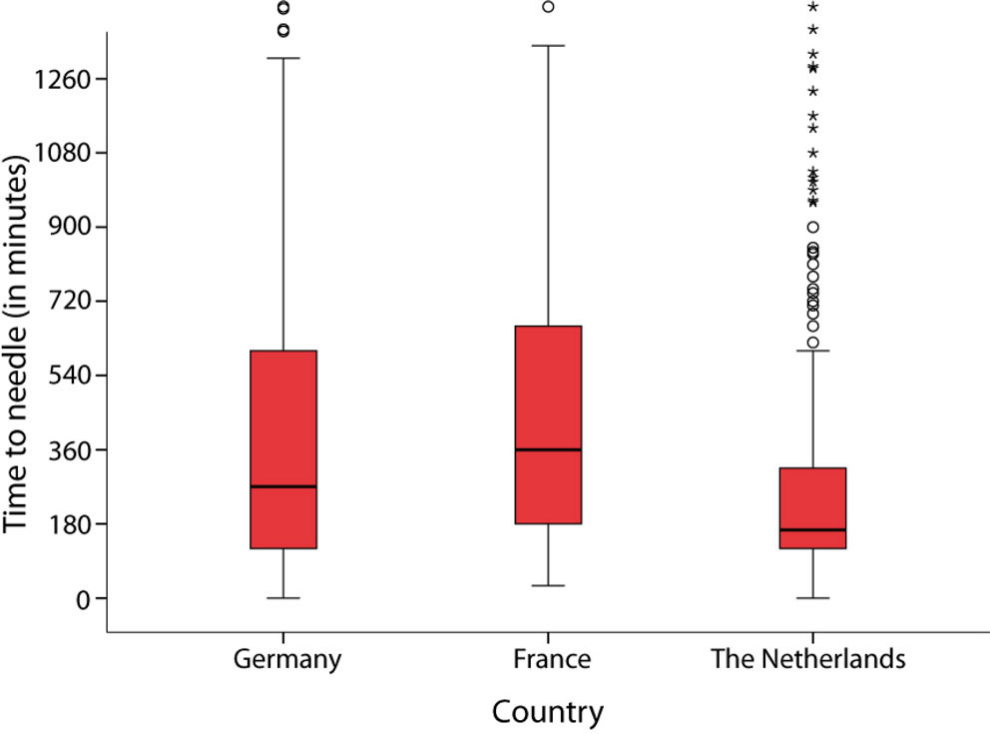
Time to needle per country

The general procedural characteristics of the study group are summarised in Table 2 and Fig. 1. There was a wide inter-country variation on symptom-to-balloon time in the APPOSITION-III trial, 165 minutes (120–318) in the Netherlands, 270 minutes (120–615) in Germany and 360 minutes (180–660) in France, respectively. At initial angiography and afterwards, TIMI flow grade 3 in the infarct-related vessel was comparable for all subgroups.

**Table 2:** Peri-procedural characteristics

Variable	Netherlands	Germany	France	*p*-value	Total study population
	*n* = 420	*n* = 165	*n* = 131		*n* = 965
Symptom onset to balloon time (min)	165 (20–318)	270 (120–615)	360 (180–660)	<0.0001	210 (120–480)
*TIMI flow culprit vessel pre-procedure*				<0.0001	–
0	229 (55 %)	92 (56 %)	92 (56 %)	–	551 (57 %)
1	54 (13 %)	33 (20 %)	33 (20 %)	–	127 (13 %)
2	78 (19 %)	27 (16 %)	27 (16 %)	–	162 (17 %)
3	54 (13 %)	13 (8 %)	13 (7.9 %)	–	120 (13 %)
*TIMI flow culprit vessel post-procedure*				0.500	–
0	4 (1.0 %)	4 (2.4 %)	0	–	10 (1 %)
1	1 (0.2 %)	1 (0.6 %)	1 (0.8 %)	–	4 (0.40 %)
2	16 (3.8 %)	8 (4.8 %)	5 (3.8 %)	–	39 (4 %)
3	397 (95 %)	152 (92 %)	125 (95 %)	–	910 (95 %)

*TIMI* Thrombolysis In Myocardial Infarction

### Medication use

The use of medication pre-hospital (in the ambulance), peri-procedural and at discharge is shown in Table 3. The Dutch patients in the APPOSITION-III trial had been preloaded with acetylsalicylic acid and a P2Y12 inhibitor, in 97 and 93 % of the cases, respectively, and with unfractionated heparin in 94 % of cases. In general, this treatment regimen was more often performed in the Netherlands and France than in Germany. The dual antiplatelet therapy at discharge was high across the whole APPOSITION-III trial.

**Table 3:** Medication use

Variable	Netherlands (%)	Germany (%)	France (%)	*p*-value	Total study population (%)
*Pre-procedure (in ambulance)*					
Acetylsalicylic acid	375/407 (92)	134/165 (81)	118/131 (90)	0.001	838/950 (88)
P2Y12 inhibitor	331/407 (81)	89/165 (54)	108/131 (82)	<0.00001	709/950 (75)
Heparin	300/407 (74)	76/165 (46)	66/131 (50)	<0.00001	574/950 (60)
*Pre- and/or peri-procedure*					
Acetylsalicylic acid	393/407 (97)	154/165 (93)	125/131 (95)	0.233	897/950 (94)
P2Y12 inhibitor	380/407 (93)	131/165 (79)	124/131 (95)	<0.00001	863/950 (91)
Heparin	381/407 (94)	154/165 (93)	89/131 (68)	<0.00001	825/950 (87)
Bivalirudin	46/407 (11)	1/165 (1)	3/131 (2)	<0.00001	109/950 (12)
Glycoprotein IIb/IIIa inhibitor	147/407 (36)	60/165 (36)	59/131 (45)	0.169	357/950 (38)
*Discharge*					
Acetylsalicylic acid	408/418 (98)	162/165 (98)	128/130 (99)	0.804	941/960 (98)
P2Y12 inhibitor	418/418 (100)	162/165 (98)	130/130 (100)	0.007	954/960 (99)
On DAPT	408/418 (98)	162/165 (98)	128/130 (99)	0.804	939/960 (98)

*DAPT* dual antiplatelet therapy

### Major adverse cardiac events and stent thrombosis

The endpoints MACE and stent thrombosis at 30 days and one-year follow-up are shown in Table 4. No important differences were seen between the different groups according these endpoints.

**Table 4:** Major adverse cardiac events and stent thrombosis at 30 days and one-year follow-up

	Netherlands		Germany		France		*p*-value	Total study population	
	*n* = 420		*n* = 165		*n* = 131			*n* = 965	
*Events at 30 days*	Patients	Event rate* (percentage)	Patients	Event rate* (percentage)	Patients	Event rate* (percentage)	–	Patients	Event rate (percentage)
MACE	18	4.3	5	3.1	0	–	0.053	34	3.5
Definite/probable ST	17	4.1	5	3.1	0	–	0.066	30	3.1
– Definite ST	15	3.6	3	1.8	0	–	0.061	24	2.5
– Probable ST	2	0.5	2	1.2	0	–	0.350	6	0.6
*Events at one-year*	*Patients*	Event rate* (percentage)	*Patients*	Event rate* (percentage)	*Patients*	Event rate* (percentage)	*–*	*Patients*	*Event rate *(percentage)
MACE	32	7.7	21	12.7	12	9.2	0.112	86	8.9
Definite/probable ST	17	4.1	6	3.6	0	–	0.070	33	3.5
– Definite ST	15	3.6	4	2.4	0	–	0.085	27	2.8
– Probable ST	2	0.5	2	1.2	0	–	0.350	6	0.6

*Kaplan-Meier estimates. *P*-value with log rank test

*MACE* major adverse cardiac events (defined as the composite of cardiac death, recurrent target-vessel related myocardial infarction and clinically-driven target lesion revascularization), *ST* stent thrombosis

## Discussion

Despite the fact that reperfusion therapies, in particular PPCI, have proven substantial effectiveness in clinical trials, worldwide mortality from STEMI outside clinical trials remains high [7]. In developing countries, both an increasing incidence of risk factors for coronary artery disease and multiple shortcomings in health policies and therapeutic options can be responsible for a higher mortality [8, 9]. Even within Europe, there is a wide inter-country variability in patient characteristics, use of medication and invasive procedures in STEMI patients [10, 11].

In 2007, for instance, only 40–45 % of European STEMI patients were treated with PPCI [12]. Especially in the southern and eastern countries thrombolysis was the preferred strategy. In northern, western and central Europe, PPCI was offered to 60–90 % of the STEMI patients [12]. Among all European countries, the Netherlands is expected to apply best-practice treatment for STEMI, because of the universal and well-developed healthcare system, the modern logistics and infrastructures, and the percentage of gross product invested in healthcare [11, 13]. Historically, the first study suggesting the advantages of PPCI vs. conventional treatment was indeed performed in the Netherlands in the early 1990 s [3]. Despite all these assets, it is at present not possible to assess national performance for STEMI in the Netherlands because a national registry is not available.

By virtue of the large number of Dutch patients enrolled, the APPOSITION-III trial offers a good opportunity for studying management of STEMI patients in the Netherlands [6], at a time when ESC STEMI guidelines had already been published.

According to the ESC STEMI guidelines, restoring coronary flow in the infarct-related vessel and myocardial tissue reperfusion are of major prognostic importance [1]. A shorter time between the occurrence of ischaemia and the achievement of reperfusion results in improved myocardial salvage and is associated with better clinical outcomes [14]. Therefore, in patients presenting with STEMI, pharmacological and/or mechanical (PCI) reperfusion should be performed as early as possible [1, 15]. Besides system delay, symptom duration may accurately reflect the risk not only of myocardial injury but also long-term mortality as suggested in a recently published analysis of the HORIZONS-AMI trial [16]. In the APPOSITION-III trial, the symptom-to-balloon time shows huge differences between the three studied cohorts and the overall population, with better performance in the Dutch cohort. These differences can only partly be explained by a more favourable geography (shorter distances) and infrastructure (more highways) in the Netherlands. Patient awareness of symptoms, fast response of the emergency services (ambulance) and direct referral to tertiary PCI-capable hospitals by an ECG transfer system (from ambulance to PCI centre) probably play a major role [13].

The results of the Dutch cohort of the APPOSITION-III trial closely resembled those of another, albeit randomised, STEMI study: the ATLANTIC trial. The ATLANTIC trial is a multicentre, international, double-arm, double-blind, randomised study comparing the uploading of ticagrelor in the ambulance or at the cathlab in STEMI patients undergoing PPCI [17]. Of the overall 1862 patients, only 7.9 % came from the Netherlands, while the rest were enrolled in 12 other European countries. The average symptom-to-balloon time of the Dutch patients in the APPOSITION-III trial is 165 minutes, which is almost similar to the 159 minutes in the ATLANTIC trial. First-medical-contact-to-balloon (FMCTB) time, i. e. the symptom-to-balloon minus the symptom-to-first-medical-contact time, which is an index of system delay, was on average 86 minutes in the ATLANTIC trial. Adams et al. [13] have reported that this is also comparable with the Dutch situation, at least for the Great Amsterdam Area. We can speculate that in the Netherlands real-life STEMI patients already receive the treatment that is recommended (Class IA) by the ESC for PPCI standard of performance (FMCTB time <90 min in 80 % of the population), while perhaps in other European countries this is only achievable in highly selected, strongly monitored randomised trials. In 2008, the ESC launched the Stent for Life initiative in order to implement effective PPCI programs and achieve the same results in all its 47 member countries across Europe and the Mediterranean basis [11].

Another important factor influencing the restoration of coronary flow in the infarct-related vessel and related myocardial tissue reperfusion is the routine use of peri-procedural dual antiplatelet (DAPT) (Class 1 A) and antithrombotic medication such as unfractionated heparin (Class 1 C). The analysis of the Dutch subgroup of the Apposition-III trial shows adequate peri- and post-procedural medical treatment in 95 %, and 98 % at discharge, respectively. In particular, the comparison of the preloading strategy with the German cohort shows important differences in the APPOSITION-III trial.

Although our retrospective analysis let some differences emerge in the management of STEMI patients between the Netherlands and other European countries, this had no significant impact on MACE at 30 days and one-year follow-up. The reason for this lack of effect is uncertain and contradictory with other previous studies, but the explanations could include a lack of power for MACE, a lack of prospectively registered surrogate outcomes and limited follow-up at this moment [13]. Most likely the higher occurrence of specific device-related events (stent thrombosis at 30 days follow-up in the APPOSITION-III was 3.1 %, while in the Atlantic was 2.2 %, for instance), as well as the disproportion in geographical inclusion, and possible selection bias in the least including countries, could have overshadowed the effect of earlier reperfusion.

Therefore, although the data from the Dutch cohort from the APPOSITION-III trial are reassuring, it will be impossible without a national STEMI registry to draw definitive conclusions on whether treatment performances are adequate for each Dutch patient presenting with STEMI. Both the National Cardiovascular Data Registry (NCDR) and the NVVC Connect projects, the first aimed at creating a national database, and the second at promoting local networks, will soon shed more light on this subject [18, 19].

## Limitations

As already mentioned, the Dutch population described in this study was retrospectively extracted from a multicentre, albeit all-comers, registry. Due to an inclusion bias (partly consecutive enrolment) and the limited amount of patients included, this study cannot be considered a true epidemiological study, but merely a snapshot with possible clinical implications. Moreover, the APPOSITION-III trial was performed in teaching and university hospitals, which may not entirely represent the reality of any country as a whole. Time to first medical contact, as well as door-to-balloon time are important parameters for quality assessment in STEMI. Neither of these data were captured in the APPOSITION-III study; therefore we can only indirectly extrapolate the Dutch performance on so-called ‘system delay’.

## Conclusion

In the Dutch cohort of an European multicentre STEMI study (APPOSITION-III trial), the performance in terms of symptom-to-balloon time, and pre-, peri- and post-procedural medical treatment matches the recommendation of ESC STEMI guidelines.

## Appendix A

### Participating centres

#### The Netherlands

- Onze Lieve Vrouwe Gasthuis (Amsterdam): 100 patients (23.8 %)
- Academic Medical Center (Amsterdam): 100 patients (23.8 %)
- Albert Schweitzer Hospital (Dordrecht): 89 patients (21.2 %)
- Erasmus Medical Center (Rotterdam): 63 patients (15 %)
- St. Antonius Hospital (Nieuwegein): 38 patients (9 %)
- VU Medical Center (Amsterdam): 17 patients (4 %)
- Scheper Hospital (Emmen): 8 patients (1.9 %)
- Amphia Hospital (Breda): 5 patients (1.2 %)

#### Germany

- SLK-Kliniken Heilbronn (Heilbronn)
- Evang. Bethesda Johanniter (Duisburg)
- Klinikum Neuperlach (Munich)
- Elisabeth-Krankenhaus (Essen)
- Klinikum Coburg
- Universitätsklinikum Giessen und Marburg
- Herz- und Diabeteszentrum NRW (Bad Oeynhausen)
- Klinikum der Universität (Munich)
- Klinikum Bogenhausen

#### France

- CHU La Pitié-Salpêtrière (Paris)
- Clinique Saint-Hilaire (Rouen)
- Institut Hospitalier Jacques Cartier (Massy)
- CHU Henri Mondor (Créteil)
- Centre Cardioloque d’Evecquemont
- Clinique de l’Europe (Amiens)
- La Roseraie (Aubervilliers)
- Centre Hospitalier Victor Dupouy (Argenteuil)
